# Structural Analysis of Glutamine Synthetase from *Helicobacter pylori*

**DOI:** 10.1038/s41598-018-30191-5

**Published:** 2018-08-03

**Authors:** Hyun Kyu Joo, Young Woo Park, Young Yoon Jang, Jae Young Lee

**Affiliations:** Department of Life Science, Dongguk University-Seoul, Ilsandong-gu, Goyang-si, Gyeonggi-do 10326 Republic of Korea

## Abstract

Glutamine synthetase (GS) is an enzyme that regulates nitrogen metabolism and synthesizes glutamine via glutamate, ATP, and ammonia. GS is a homo-oligomeric protein of eight, ten, or twelve subunits, and each subunit-subunit interface has its own active site. GS can be divided into GS I, GS II, and GS III. GS I and GS III form dodecamer in bacteria and archaea, whereas GS II form decamer in eukaryotes. GS I can be further subdivided into GS I-α and GS I-β according to its sequence and regulatory mechanism. GS is an essential protein for the survival of *Helicobacter pylori* which its infection could promote gastroduodenal diseases. Here, we determined the crystal structures of the GS from *H*. *pylori* (*Hpy* GS) in its apo- and substrate-bound forms at 2.8 Å and 2.9 Å resolution, respectively. *Hpy* GS formed a dodecamer composed of two hexameric rings stacked face-to-face. *Hpy* GS, which belongs to GS I, cannot be clearly classified as either GS I-α or GS I-β based on its sequence and regulatory mechanism. In this study, we propose that *Hpy* GS could be classified as a new GS-I subfamily and provide structural information on the apo- and substrate-bound forms of the protein.

## Introduction

In gram-negative bacteria, ammonia is a unique molecule required for nitrogen anabolism. Nitrogen is essential for the synthesis of key constituents of the cell, such as amino acids, NAD, pyrimidines, purines, and amino sugars^1^. In general, ammonia assimilation in bacteria is associated with three enzymes: glutamine synthetase (GS), glutamate synthase (GOGAT), and glutamate dehydrogenase (GDH)^2^. Nitrogen starvation leads to GS-GOGAT mediated glutamine synthesis, whereas high ammonia availability leads to the activation of GDH^3,4^. The pathways involved in ammonia assimilation are common among bacteria, cyanobacteria, algae, yeasts, and fungi^5^. In *Helicobacter pylori*, ammonia is produced by urease, which exhibits higher activity compared with that in other species, and is used in the neutralization of gastric acid for survival in animal hosts^6^. The remaining ammonia is then processed by GS. The GS in *H*. *pylori* (*Hpy* GS) is a critical enzyme that is involved in processing the ammonia released by urease activity, and nitrogen metabolism^7^.

GS is an enzyme involved in the biosynthetic reaction producing glutamine from the condensation of glutamate and ammonia^8^. This enzyme is present in all living organisms and is a large homo-oligomeric complex composed of eight, ten, or twelve subunits assembled into two face-to-face rings^9^. The catalytic and regulatory loops of all GS enzymes including *Hpy* GS are well conserved, resulting in a similar biosynthetic mechanism. Each active site at the subunit-subunit interface has two main entrances for ATP and glutamate^10^. The active site of GS is generally described as a ‘bifunnel’, where the entrance for ATP is located at the top of the bifunnel opening to the external surface, and glutamate can enter through the bottom of the bifunnel. With the presence of divalent cations such as magnesium or manganese ions, the carboxyl oxygen in the side chain of glutamate attacks the terminal phosphorus of ATP, resulting in a γ-glutamyl phosphate intermediate. The intermediate is subsequently attacked by ammonia to form glutamine^11,12^.

The GS in all living organisms can be categorized into three different classes: GS I, GS II, and GS III^13^. GS I and GS III enzymes are found in bacteria and archaea and mostly form a dodecamer, whereas GS II enzymes are found in eukaryotes and form a decamer^14,15^. GS I enzymes are further subdivided into two GS isoenzymes: GS I-α and GS I-β. GS I-α enzymes are generally found in low G + C gram-positive bacteria, thermophilic bacteria, and euryarchaeota, whereas GS I-β enzymes are found in other bacteria species^16,17^. There are two distinct differences between GS I-α and GS I-β enzymes. GS I-α enzymes are mainly feedback-inhibited by the end products of glutamine metabolism including AMP and glutamine, whereas GS I-β enzymes are additionally inactivated by the adenylation of a tyrosine residue near the active site (NL**Y**DLP). Furthermore, the insertion of a specific 25-amino acid residues (residues 146–170 in *Salmonella typhimurium* GS) occurs in GS I-β but not GS I-α^13^. However, *Hpy* GS is unique and does not fit into either subdivision. Although the structural and sequence information of *Hpy* GS indicate its relationship with GS I-β due to the presence of a specific 25-amino acid residues (residues 155–179), the adenylation site (NL**Y**DLP) found in similar homologs is replaced in *Hpy* GS (NL**F**^**407**^KLT), suggesting a lack of the adenylation regulatory mechanism in *Hpy* GS.

Several crystal structures of GS from bacterial species including *Mycobacterium tuberculosis*, *Salmonella enterica*, *Bacillus subtilis*, and *Bacteroides fragilis* have been determined^18–21^. The GS from *B*. *subtilis* (*Bsu* GS) is categorized as GS I-α, which lacks both the adenylation regulatory mechanism and the specific 25-amino acid residues^22,23^. Structural analysis of *Bsu* GS has revealed that it has a unique feedback inhibition mechanism distinct from that of GS I-β enzymes^20^. The GS from *S*. *typhimurium* (*Sty* GS), which is categorized as GS I-β, is subjected to adenylation on the tyrosine residue in NLY^407^DLP and has the specific 25-amino acid residues^24^. The crystal structures of *Sty* GS reports that a flexible loop (residues 324–329 in *S*. *typhimurium*) near the active site protects the intermediate and completes the ammonium binding site upon ATP and glutamate binding^12^.

*Hpy* GS is a critical enzyme required for the survival of *H*. *pylori* in animal hosts and may be used to target *H*. *pylori*, which causes duodenal ulcers and stomach cancer in humans^7^. Structural information on *Hpy* GS will be valuable for designing new selective inhibitors. We performed crystallographic analysis to further understand the structural and regulatory features of *Hpy* GS, which cannot be classified as either GS I-α or GS I-β. Here, we report the *Hpy* GS structures of the apo-, substrate-bound form, and the intermediate state. The results demonstrated the formation of *Hpy* GS from a dimer of hexameric rings, and a comparison of the apo-, substrate-bound form, and intermediate state revealed the catalytic mechanism upon substrate binding.

## Results

### Structure determination and model quality

To determine the structure of *Hpy* GS, crystals of the apo- and substrate-bound forms in complex with ATP and phosphinothricin (PPT) were obtained. The apo structure of *Hpy* GS (*Hpy* GS^apo^) was determined at 2.8 Å by molecular replacement using the GS model from *S*. *typhimurium* (1F52) and refined to crystallographic *R*_work_ and *R*_free_ values of 18.99% and 26.01%, respectively. The refined model (PDB entry 5ZLI) contained 2,846 amino acid residues of six identical subunits in the asymmetric unit, which could be used to generate a dodecamer. In each subunit, N-terminal residues (Met1–Asn7 in subunit A, E, and F; Met1–Gln6 in subunit B, C, and D) and internal residues (Leu408–Gly418 in all subunits) were disordered.

The substrate-bound form of *Hpy* GS (*Hpy* GS^sub^) was obtained in the presence of ATP and PPT, which is a structural analogue of glutamate. *Hpy* GS^sub^ was determined at 2.9 Å and refined to crystallographic *R*_work_ and *R*_free_ values of 16.45% and 24.27%, respectively. The refined model (PDB entry 5ZLP) contained 5,704 amino acid residues of twelve identical subunits in the asymmetric unit. In each subunit, N-terminal residues (Met1–Thr5 in subunit A, D, G, and L; Met1–Gln6 in subunit B, F, H, and K; Met1–Asn7 in subunit C; Met1–Ser8 in subunit E; Met1–Ile2 in subunit I; Met1–Val3 in subunit J) and internal residues (Leu408–Gly418 in all subunit) were disordered. Model qualities and refinement statistics are summarized in Table 1.

**Table 1:** Data collection and refinement statistics.

	Apo	ATP + phosphinothricin
**Data collection statistics**		
Wavelength (Å)	0.97950	0.97950
Resolution (Å)^a^	50.00–2.80 (2.85–2.80)	50.00–2.90 (2.95–2.90)
Space group	C 2	C 2
Unit-cell parameters (Å,°)	a = 191.09, b = 131.46, c = 131.41,	a = 234.46, b = 135.20, c = 203.09,
	α = 90.00, β = 122.68, γ = 90.00	α = 90.00, β = 91.59, γ = 90.00
Number of observations	224008	454812
Unique reflections	67460	135884
Data completeness (%)	99.5 (99.8)	98.8 (99.7)
Redundancy	3.3 (3.4)	3.3 (3.4)
Average I/σ(I)	10.3 (2.5)	5.4 (3.8)
R_merge_ (%)^b^	14.0 (51.3)	17.7 (43.4)
**Refinement statistics**		
R_work_/R_free_ (%)	18.99/26.01	16.45/24.27
rmsd bonds (Å)	0.01	0.01
rmsd angles (°)	1.28	1.12
Average *B* factors (Å^2^)	43.91	29.84
**Ramachanran Plot (%)**		
Favored	93.82	91.43
Allowed	5.51	7.9
Outliers	0.67	0.67

^a^Numbers in parentheses reflect the highest resolution shell. ^b^*R*_merge_ = Σ_h_Σ_i_|*I*(*h*)_i_ − < *I*(*h*) > |/Σ_h_Σ_i_*I*(*h*)_i_, where *I*(*h*) is the intensity of reflection *h*, Σ_h_ is the sum over all reflections, and Σ_i_ is the sum over i measurements of reflection *h*.

### Overall structure of *Hpy* GS

**Figure 1:**
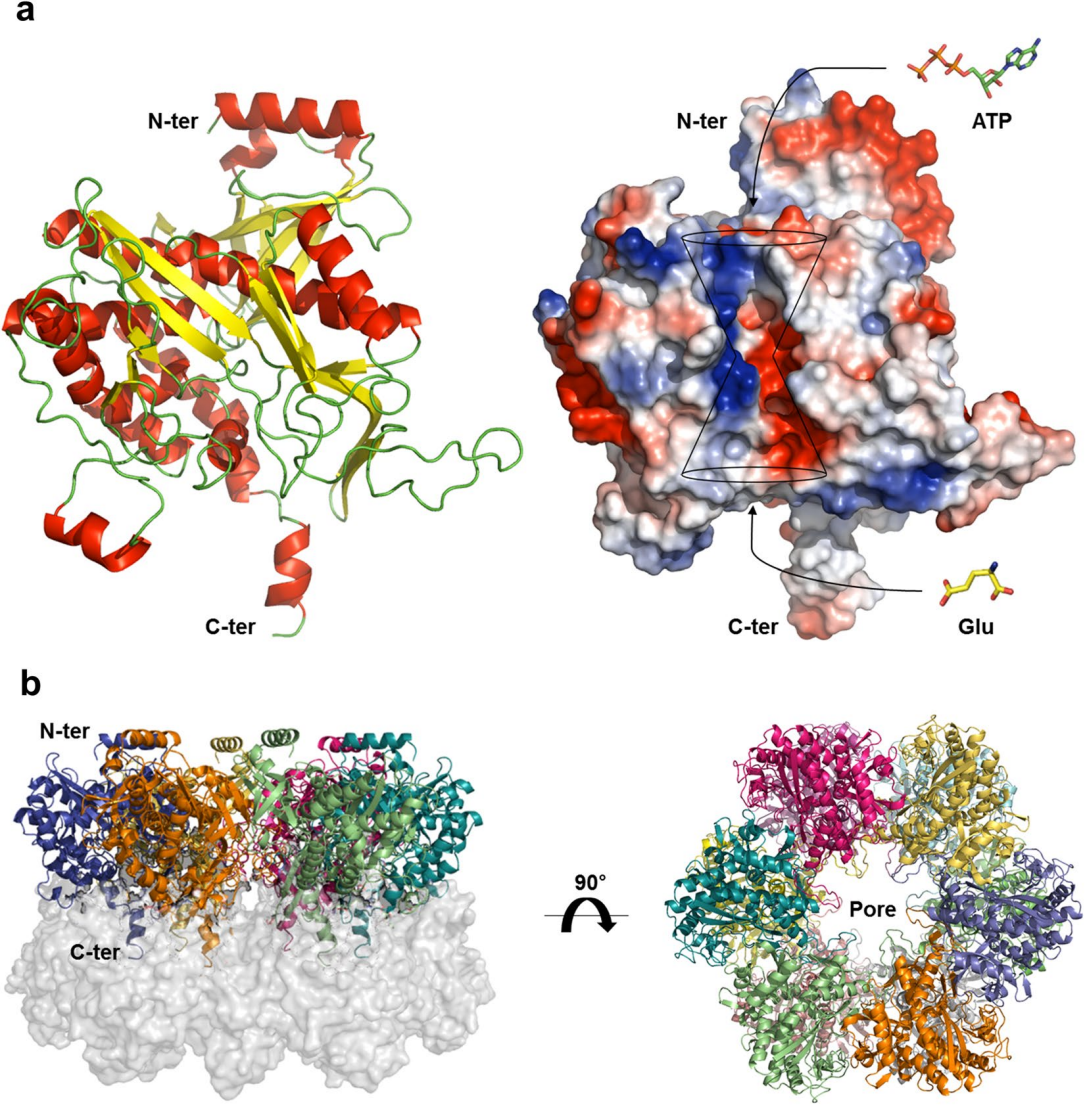
Overall structure of GS from *H*. *pylori*. (**a**) The monomeric structure of *Hpy* GS^apo^. Each active site has an entrance for ATP and glutamate. (**b**) Oligomeric structure of *Hpy* GS^apo^ was generated by two-fold symmetry from hexamer in an asymmetric unit. The C-terminal helical thong of each subunit interacted with the C-terminal thong of opposite subunits. Twelve active sites were positioned at each subunit-subunit interface.

**Figure 2:**
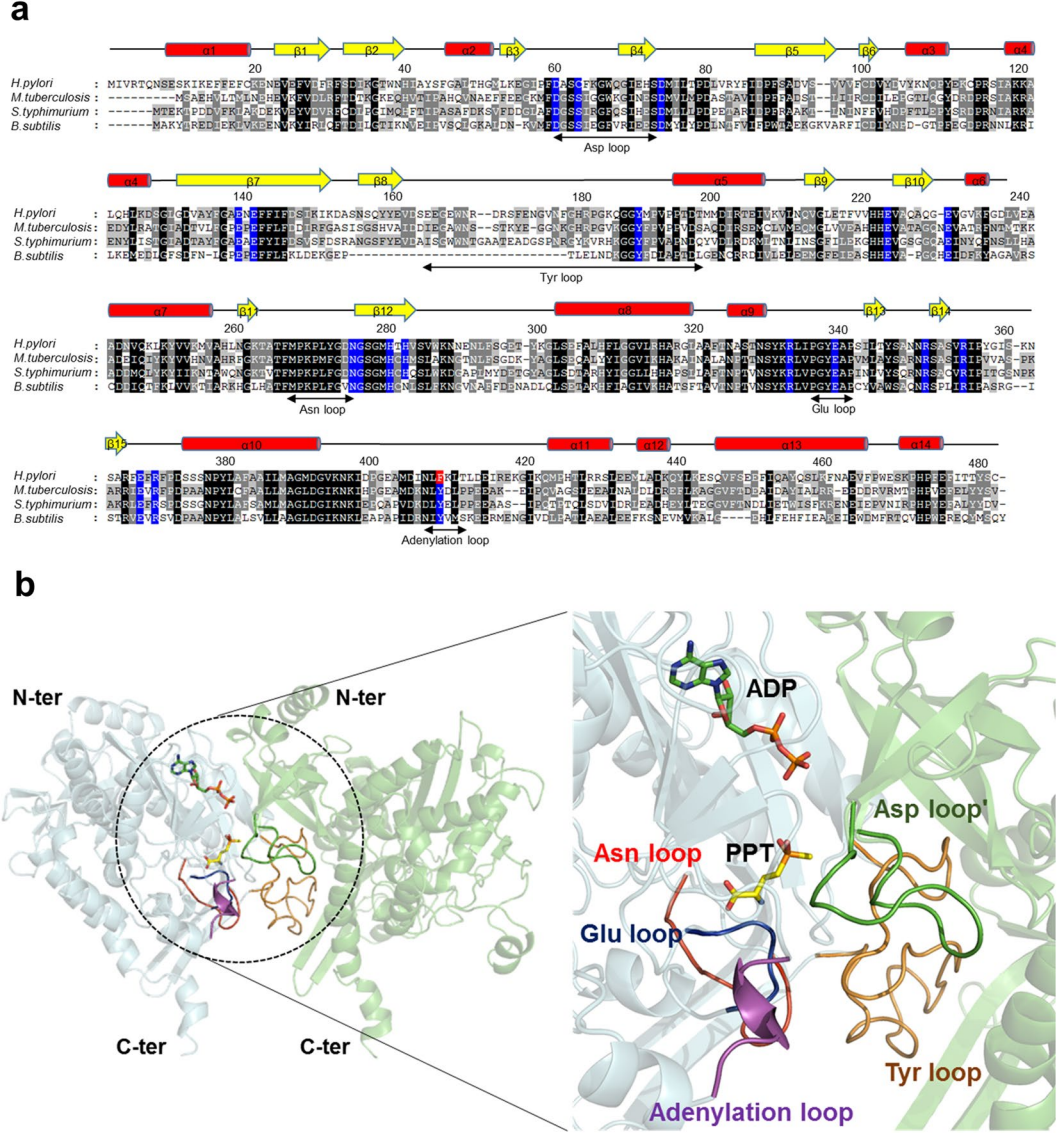
Catalytic and regulatory loops of *Hpy* GS. (**a**) Multiple sequence alignment of *Hpy* GS with other homologous GS sequences in bacteria. The highly conserved residues are indicated by black boxes. The active site and catalytic residues are indicated by blue boxes. The tyrosine residue replaced to phenylalanine in *Hpy* GS is coloured in red box. (**b**) The catalytic and regulatory loops were well conserved, completing the active site between the subunit-subunit interfaces. The Asp loop was contributed from the neighbouring subunit.

The monomeric structure of *Hpy* GS containing 481 amino acid residues was composed of 14 α-helices and 15 β-strands, which could be divided into the N-terminal domain (residues 1–113) and C-terminal domain (residues 114–481) (Fig. 1a). The N-terminal domain was exposed to the solvent, whereas the C-terminal helix, called the ‘helical thong’, was inserted into a hydrophobic hole in the opposite subunit of the other hexameric ring (Fig. 1b). The catalytic and regulatory loops of GS were highly conserved, which included the Glu loop (flap, PGY**E**^**337**^AP), Asp loop (latch, **D**^**60**^–D^74^), Asn loop (residues F^265^–**N**^**274**^), Tyr loop (S^163^–**Y**^**190**^–M^199^), and adenylation loop (NL**F**^**407**^KLT)^9,12^ (Fig. 2a,b). These loops completed the classical ‘bifunnel’ structure of the active site. In general, the adenylation loop is located near the active site and regulates GS activity by adenylation^13^. Although the location of the adenylation loop of *Hpy* GS was conserved with that of other GS enzymes, the tyrosine residue (NL**Y**DLP) of the adenylation site was replaced with phenylalanine (NL**F**^**407**^KLT) (Fig. 2a). As a result, *Hpy* GS could not be regulated by adenylation which is a unique feature among GS I-β subfamily members.

### Oligomeric structure of *Hpy* GS

Although a hexamer of *Hpy* GS^apo^ was present in each asymmetric unit of the crystal, it could be used to generate a dodecamer with 62 symmetry by stacking two hexameric rings face-to-face (Fig. 1b). The hexameric structure of *Hpy* GS was formed mainly by the Tyr loop of each subunit interacting through hydrogen bonds, hydrophobic interactions, and salt bridges. The solvent-accessible surface area (SAS) buried at the interface between the subunits in the hexameric structure of *Hpy* GS^apo^ and *Hpy* GS^sub^ were calculated to 3,698 Å^2^ and 3,672 Å^2^ (~18% of the monomer surface area), respectively (Protein–Protein Interaction Server PDBePISA at http://www.ebi.ac.uk/msd-srv/prot_int/). The hexameric interface of the dodecamer was composed of C-terminal helices 13 and 14 (residues 461–481), which extended to a hydrophobic hole of the opposite hexameric ring. The C-terminal helices 13 and 14 of one hexamer was interlocked with the C-terminal helices of the opposite hexamer via hydrophobic interactions (A461, F464, P465, W466, P472, F473, F475, and I476) and hydrogen bonds (E462 Oε1 to T478 Oγ1, K469 O to H471 Nε2, P470 O to H471 Nε2, H471 Nε2 to K469 O, H471 Nε2 to P470 O, E474 Oε1 to T326 Oγ1, E474 Oε1 to K330 Nζ, E474 Oε2 to N327 Nδ2, T477 O to K35 Nζ, T478 Oγ1 to E462 Oε2, Y479 OH to T262 Oγ1, Y479 OH to A263 N, S480 O to L253 Nζ, and C481 Sγ to S373 O) (Fig. S1). The SAS buried at the interface between the hexameric rings in the dodecameric structure of *Hpy* GS^apo^ and *Hpy* GS^sub^ were calculated to 2,875 Å^2^ and 2,870 Å^2^ (~13% of the monomer surface area), respectively. These results demonstrated the dodecameric form of *Hpy* GS.

### Structural comparisons

Six subunits in the asymmetric unit of *Hpy* GS^apo^ were almost identical to each other with root-mean-square deviation (r.m.s.d.) values of 0.22–0.27 Å for 474 Cα atoms in residues 8–481. In *Hpy* GS^sub^, twelve subunits in the asymmetric unit were also highly similar with r.m.s.d. values of 0.29–0.36 Å for 475 Cα atoms in residues 7–481. Superposition of *Hpy* GS^apo^ and *Hpy* GS^sub^ gave a r.m.s.d. value of 0.50 Å for 473 Cα residues (residues 9–481), and a r.m.s.d. value of 0.50 Å for 4,742 Cα atoms for dodecamer-dodecamer comparisons. The r.m.s.d. values of the catalytic and regulatory loops were calculated and ranged from 0.45 Å to 1.1 Å (Table S1.). The adenylation loops had higher r.m.s.d. values, which may be attributed to the disorder of the loop. These results indicated that *Hpy* GS does not undergo a large conformational change upon substrate binding.

### Active site of *Hpy* GS

**Figure 3:**
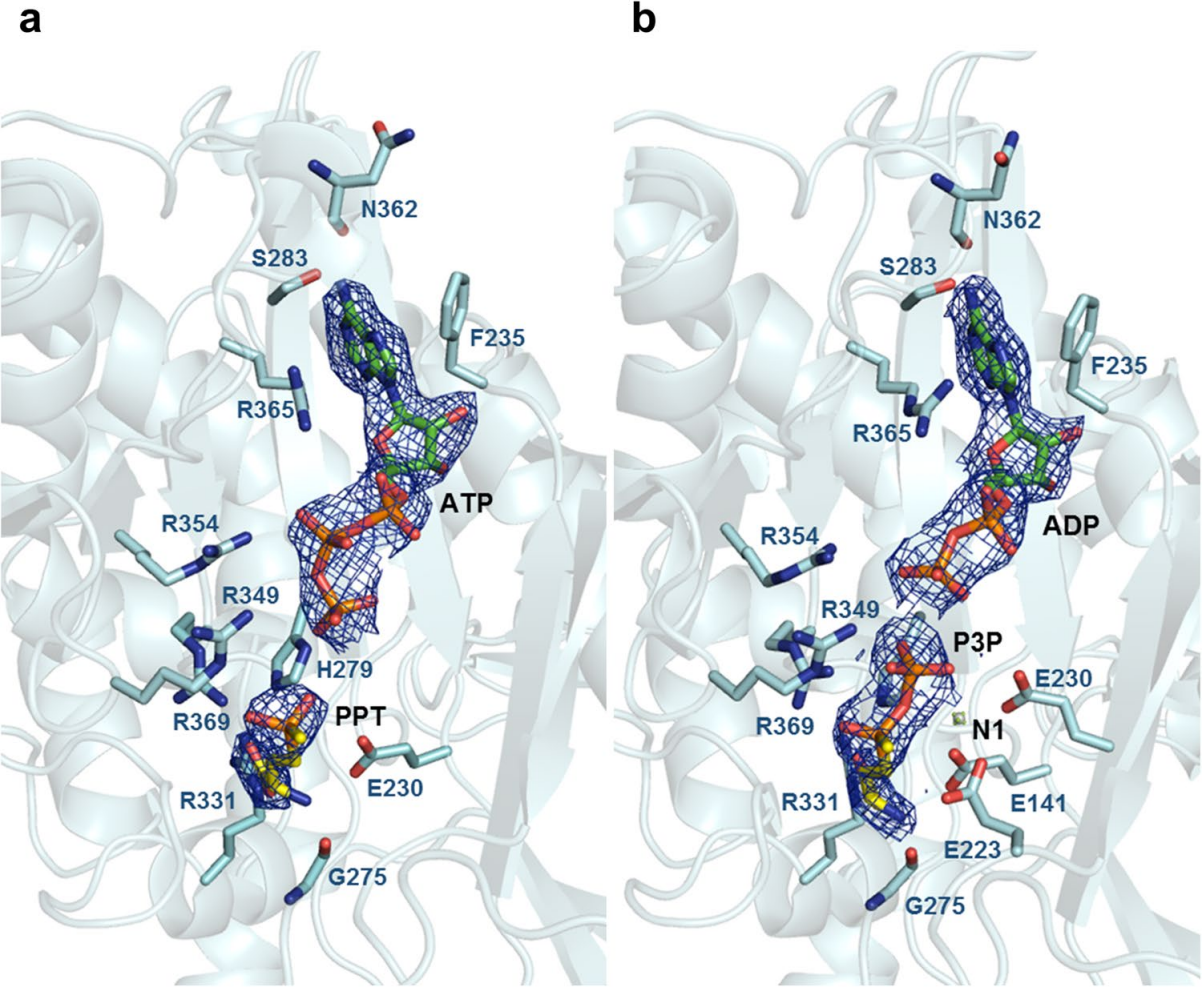
Active site of *Hpy* GS. (**a**) The active site of *Hpy* GS^sub^ with ATP and PPT. (**b**) The active site of *Hpy* GS^int^ with ADP and P3P. The N, H, and O atoms of the ligands are coloured in blue, grey, and red, respectively. The C atoms of *Hpy* GS, ATP/ADP, and PPT/P3P are presented in light blue, green, and yellow, respectively. The 2*Fo-Fc* maps of ATP/ADP and PPT/P3P are shown.

The active site and substrate-binding residues of *Hpy* GS were examined using ATP and PPT as substrates. The active site was located at the centre of the bifunnel contributed by the Glu loop (flap, residues 334–339), Asn loop (residues 265–277), and Tyr loop (residues 163–199) of each subunit and the Asp loop (latch, residues 60–74) of the neighboring subunit (Fig. 2b). As shown in other GS structures, the entrance for ATP was located at the top of the bifunnel opening to the external surface, and glutamate could enter through the bottom of the bifunnel facing the hexameric interface (Fig. 1a). In general, PPT is phosphorylated in GS in the presence of ATP and forms an intermediate state analogue, phosphinothricin phosphate (P3P), and ADP^24,25^. However, although ATP and PPT were added to *Hpy* GS, ATP/ADP substrates were clearly identified in all subunits, whereas PPT/P3P substrates were not identified in most of the subunits of *Hpy* GS. Among twelve subunits, PPT was not phosphorylated, resulting in a substrate-bound form with PPT and ATP in three subunits. In only one subunit, PPT was phosphorylated by ATP hydrolysis, resulting in an intermediate state with P3P and ADP (Fig. 3a). Thereafter, we described the *Hpy* GS bound to P3P and ADP as intermediate state (*Hpy* GS^int^).

All known 19 active site and catalytic residues in bacterial GS^9^ were mostly well conserved in *Hpy* GS except for a serine residue (S53 in *S*. *typhimurium* and S57 in *M*. *tuberculosis*), which was replaced with cysteine (C63 in *H*. *pylori*). This suggests the similarity of the overall catalytic mechanism of the *Hpy* GS with that of other bacterial GS proteins. The adenine ring of ATP/ADP was oriented similarly to *Mtb* GS^26^, bound by hydrogen bonds (N1 to S283 Oγ, and N6 to N362 O), and stacked in the hydrophobic patch by residues F235 and R365. The phosphate group of ATP/ADP was bound by ionic interactions (O1α to R365 NH2, and O2β to R349 NH2). PPT was bound by six hydrogen bonds with *Hpy* GS (NP to E141 Oε1/G275 O, OTP to R331 Nε, OP to H279 Nε2/R331 NH2, and OεB to R369 NH2), and P3P was bound by seven hydrogen bonds, where the O13 atom additionally formed a hydrogen bond with R349 NH2 (Fig. 3a,b). The possible ammonium binding site was blocked by the methyl group of P3P, acting as an inhibitor.

Two or three divalent metal ions have been reported to be involved in the active site of GS in other organisms. Two metal ions (n1 and n2 metal-binding sites) were identified per subunit in the substrate-bound GS from *S*. *typhimurium*, and three metal ions (n1, n2, and n3 metal-binding sites) were identified in the substrate-bound GS from *M*. *tuberculosis*. In both the *Hpy* GS^int^ and *Hpy* GS^sub^ structures, one magnesium ion was identified in the n1 metal binding site. The magnesium ion was coordinated with P3P (OεB and O15), E141 (Oε1), E230 (Oε2), and E223 (Oε1) (Fig. S2).

### Comparison with other GS proteins

The structural similarity of *Hpy* GS with other known bacterial GS proteins was determined by DALI server^27^. The most similar structure was the *Mtb* GS (GS I-β, 3ZXR) with a r.m.s.d. value of 1.2 Å for 476 equivalent Cα atoms and a Z-score of 54.3. The *Sty* GS (GS I-β, 1FPY) was the next most similar structure with a r.m.s.d. value of 1.2 Å for 467 equivalent Cα atoms and a Z-score of 53.9. A comparison of *Hpy* GS with the *Bsu* GS (GS I-α, 4LNI) revealed a r.m.s.d. value of 1.6 Å for 432 equivalent Cα atoms and a Z-score of 48.2. The overall structural comparison of *Hpy* GS with GS I-α and GS I-β revealed high structural similarities (Fig. S3).

**Figure 4:**
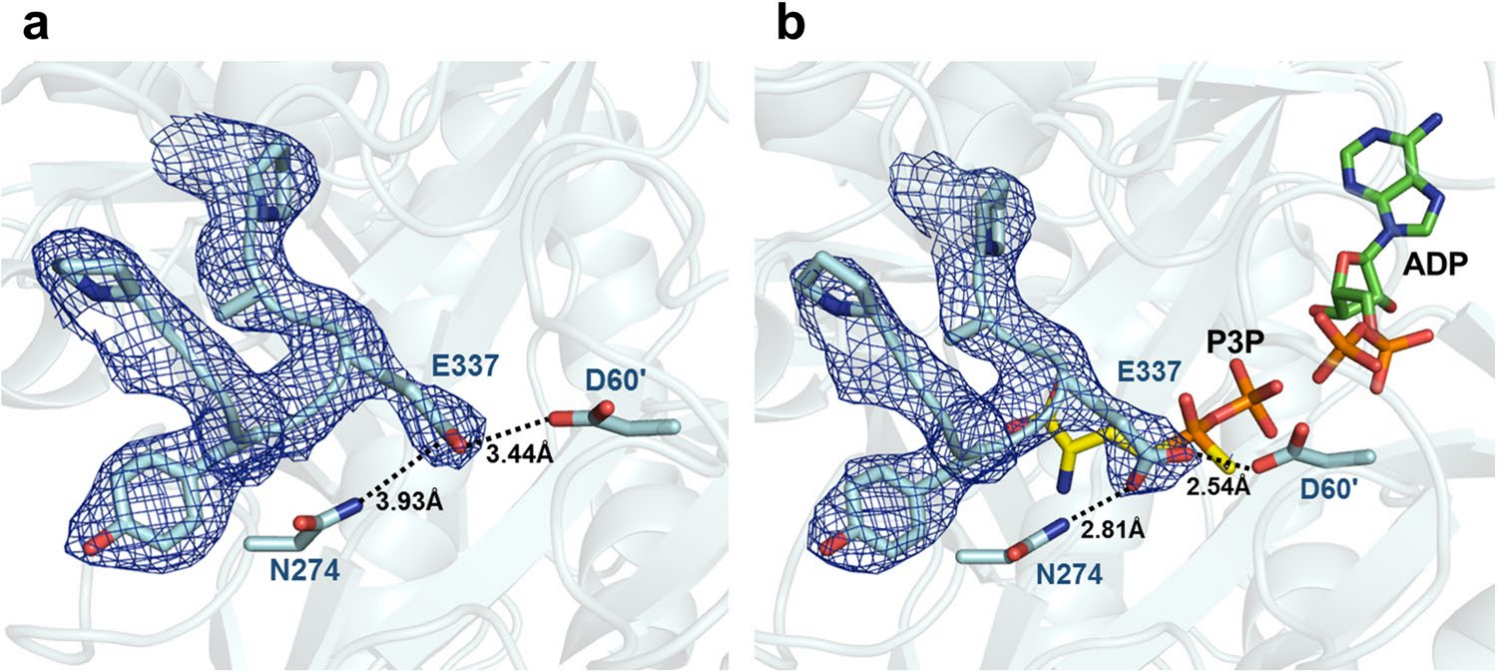
Entrance for glutamate in *Hpy* GS. (**a**,**b**) Show the entrance for glutamate in *Hpy* GS^apo^ and *Hpy* GS^int^, respectively. The N, H, and O atoms of the ligands are coloured in blue, grey, and red, respectively. ADP and P3P are represented in green and yellow, respectively. Black dotted lines denote hydrogen bonds.

Earlier studies of the GS from *S*. *typhimurium*, *M*. *tuberculosis*, and *B*. *subtilis* have demonstrated a structural change between the apo- and substrate-bound forms. The Glu flap in these GS structures (residues 324–329 in *S*. *typhimurium*, 324–329 in *M*. *tuberculosis*, and 301–306 in *B*. *subtilis*) was a flexible loop, which contributed to the entrance of the glutamate binding site with a conformational change between an open and closed form^12,19^. However, the Glu flap in both the *Hpy* GS^apo^ and *Hpy* GS^sub^ structures showed a similar closed conformation with little differences. The side chain of E337 in the *Hpy* GS^apo^ structure was disordered, demonstrating the flexibility of the Glu flap, whereas the side chain of E337 in the *Hpy* GS^sub^ structure formed strong hydrogen bonds with the N274 Oδ1 and D60 Nδ2 of the adjacent subunit (Fig. 4a,b). This finding suggests that the Glu flap in *Hpy* GS^apo^ could exist in the open or closed form in the absence of substrates.

## Conclusions

We determined the apo- and substrate-bound forms of *Hpy* GS by X-ray crystallography. A comparison of *Hpy* GS with *Sty* GS, *Mtb* GS, and *Bsu* GS also revealed high similarities in the structure and regulatory amino acid residues. However, the open form of GS was not found in *Hpy* GS, and the Glu flap was well ordered with a closed conformation in *Hpy* GS^apo^ in the absence of substrates. This suggests that *Hpy* GS could exist in either the open or closed form regardless of the presence of substrates. Based on the sequence and structural information of *Hpy* GS, this protein cannot be clearly classified as GS I-α or GS I-β. Therefore, we may classify *Hpy* GS as a new member of the GS I subfamily, which has a specific 25-amino acid sequence and lacks the adenylation regulatory mechanism. As GS is a critical enzyme required for the survival of *H*. *pylori*, structural studies of *Hpy* GS can provide further insight into drug development for the treatment of *H*. *pylori* infection.

## Methods

### Expression and purification

The gene encoding GS (*glnA*) was amplified by polymerase chain reaction using the genomic DNA of *H*. *pylori* as a template. It was inserted into the Nde1/Xho1-digested expression vector pET28b(+) (Novagen, Germany), containing a hexahistidine tag at its N-terminus. The recombinant *Hpy* GS was transformed and expressed in *E*.*coli* BL21 (DE3) star pLysS cells (Invitrogen, USA). The cells harbouring the *glnA* gene were grown at 310 K to an OD_600_ of ~0.5 in Luria-Bertani medium supplemented with 30 µg mL^−1^ kanamycin and chloramphenicol. Overexpression of the recombinant protein was induced with 1.0 mM isopropyl β-D-thiogalactopyranoside (IPTG), and cell growth was continued at 303 K for 4 h. The cells were harvested by centrifugation at 4,200 *g* for 10 min at 277 K and immediately frozen at 193 K. Cell pellets were resuspended in lysis buffer [20 mM Tris–HCl pH 8.0/0.5 M NaCl/10% (v/v) glycerol/1 mM phenylmethylsulfonylfluoride] and lysed using an ultra sonicator (Sonics^TM^ Vibra Cell VCX 750; Sonics, USA). The insoluble fractions were removed by centrifugation at 31,000 g for 1 h at 277 K.

The recombinant *Hpy* GS in the supernatant fraction was loaded on a nickel-charged His-trap immobilized metal affinity chromatography (IMAC) column (GE Healthcare, UK). The hexahistidine tagged *Hpy* GS was loaded with buffer A [20 mM Tris-HCl pH 8.0/500 mM NaCl/10% (v/v) glycerol] and eluted by buffer B [20 mM Tris-HCl pH 8.0/500 mM NaCl/10% (v/v) glycerol/300 mM imidazole]. The eluted proteins were loaded in a Superdex 200 gel-filtration column (GE Healthcare, UK) for further purification with elution buffer [20 mM Tris-HCl pH 8.0/200 mM NaCl/5% (v/v) glycerol/1 mM dithiothreitol (DTT)/2 mM MgCl_2_].

### Crystallization

Purified *Hpy* GS was concentrated to 41 mg mL^−1^ using Centricon YM-10 (Millipore, USA) for crystallization trials. Initial screening was performed by the sitting-drop vapour diffusion method using 96-well CrystalQuick plates (Greiner Bio-One, Germany) with various commercial screens (Hampton Research, USA; Qiagen, Germany; Axygen, USA; Emerald Biosystems, USA). Each sitting drop was prepared by mixing 0.75 µl protein solution and 0.75 µl reservoir solution and incubated at room temperature. The initial crystals of *Hpy* GS were grown after four weeks of incubation under several conditions with polyethylene glycol (PEG) 6,000. The crystals of *Hpy* GS were further optimized, and the best crystals were grown in 10% (v/v) PEG 6,000, 100 mM HEPES pH 7.0, and 50 mM choline. To obtain the cocrystals with substrates, *Hpy* GS was concentrated to 19 mg mL^−1^ and mixed with 5 mM ATP, 5 mM PPT, and 5 mM magnesium chloride before crystallization. It was stored at 277 K for 1 h. The crystals of substrate-bound *Hpy* GS were grown in 2.0 M sodium formate and 100 mM sodium citrate at pH 5.0.

### X-ray data collection

Crystals of the apo- and substrate-bound *Hpy* GS were transferred to a cryoprotectant solution containing 30% PEG 6,000 and 30% glycerol in reservoir solution, respectively, and immediately flash-cooled in liquid nitrogen. X-ray diffraction data were collected at 100 K on an ADSC Quantum 315 CCD image-plate detector using synchrotron radiation on beamline 5 C of the Pohang Accelerator Laboratory, Pohang, Republic of Korea. Data were collected with 1° oscillation per image and a crystal-to-detector distance of 650 mm, and a total of 180 frames were recorded. Data were processed and scaled using the *HKL*-2000 program suite^28^.

### Structure determination and refinement

The crystal structure of the apo form of *Hpy* GS was solved by molecular replacement using the GS structure from *S*. *typhimurium* as a template (1F52) with PHASER from the CCP4 program suite. *Hpy* GS in complex with ATP and PPT was solved using the apo form of *Hpy* GS as a template in the same program suite. Both structures were refined with REFMAC from the CCP4 program suite with intensity based twin refinement. Further refinement was carried out by the COOT and PHENIX program package. The refined model was finally evaluated by MolProbity. Data collection and refinement statistics are presented in Table 1.

### Accession numbers

Coordinate and structure factors have been deposited in the Protein Data Bank (PDB): apo *Hpy* GS, PDB ID, 5LZI; substrate-bound *Hpy* GS, PDB ID, 5ZLP.

## Electronic supplementary material

Supplementary Information
