# Supplementary material for: Structural Analysis of Glutamine Synthetase from Helicobacter pylori
Source: Sci Rep. 2018 Aug 3;8:11657. doi: 10.1038/s41598-018-30191-5 (PMC6076278; doi:10.1038/s41598-018-30191-5)
Supplement: Supplementary file 1 — Supplementary Information [file 41598_2018_30191_MOESM1_ESM.pdf]

Structural Analysis of Glutamine Synthetase from *Helicobacter pylori*

Hyun Kyu Joo[1], Young Woo Park[1], Young Yoon Jang[1] and Jae Young Lee[1]*

[1]Department of Life Science, Dongguk University-Seoul, Ilsandong-gu, Goyang-si, Gyeonggi-do, 10326, Republic of Korea

*Correspondence and requests for materials should be addressed to J.Y.L. (email: jylee001@dongguk.edu)

Supplementary Table 1. The r.m.s.d. values of each catalytic and regulatory loops in *Hpy* GS.

	Hpy GS^{apo} (Å)	*Hpy* GS^{sub} (Å)	*Hpy* GS^{int} (Å)	Glu loop (Å) (PGY**E**^{337}AP)	Asp loop (Å) (**D**^{60}-D^{74})	Asn loop (Å) (G^{265}-**N**^{274})	Tyr loop (Å) (S^{163}-**Y**^{190}-M^{199})	Adenylation loop (Å) (NL**F**^{407}KLT)
***Hpy* GS^{apo}**		0.50	0.50	0.68[a]	0.59[a]	0.51[a]	0.53[a]	1.0[a]
***Hpy* GS^{sub}**	0.50		0.32	0.47[b]	0.55[b]	0.45[b]	0.50[b]	0.60[b]
***Hpy* GS^{int}**	0.50	0.32		0.61[c]	0.60[c]	0.50[c]	0.60[c]	1.1[c]

[a]R.m.s.d. between *Hpy* GS^{apo} and *Hpy* GS^{sub}

[b]R.m.s.d. between *Hpy* GS^{sub} and *Hpy* GSint

[c]R.m.s.d. between *Hpy* GS^{apo} and *Hpy* GSint

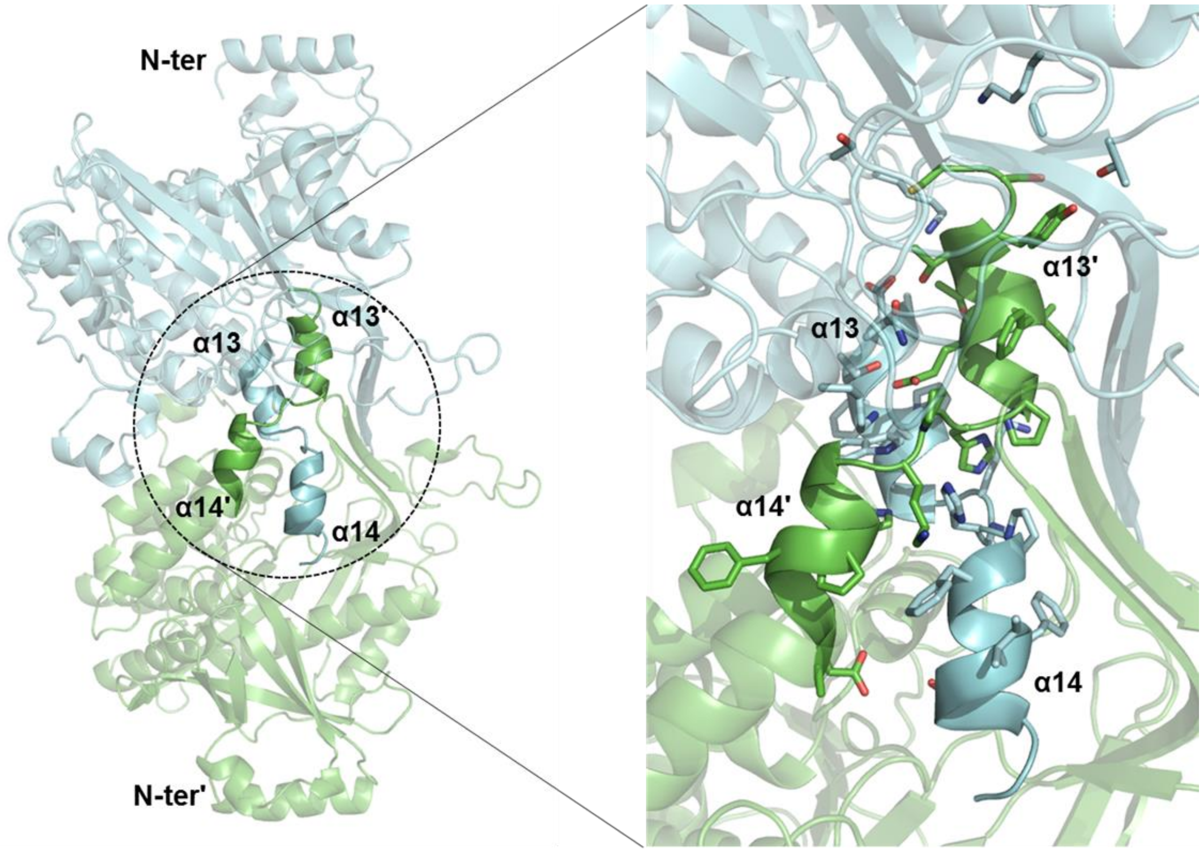

Supplementary Figure 1. Hexamer to hexamer interactions of *Hpy* GS. The C-terminal helix (helical thong) in *Hpy* GS is shown. The helical thong is extended and inserted into the opposite hexameric ring. N, H, and O atoms of the ligands are coloured in blue, grey, and red, respectively.

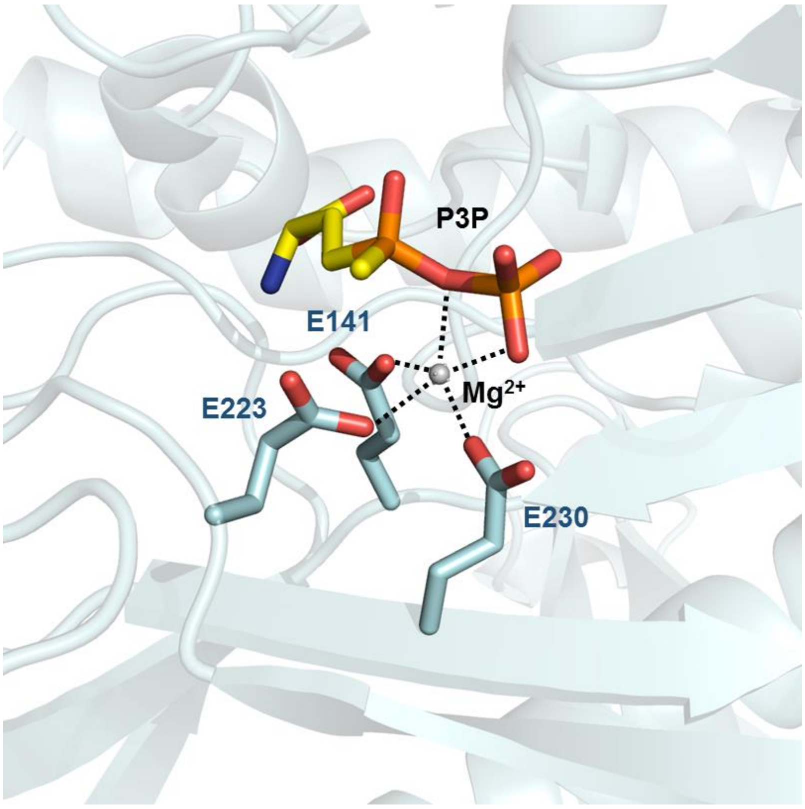

Supplementary Figure 2. Metal binding site of *Hpy* GS[int]. The N, H, and O atoms of the ligands are coloured in blue, grey, and red, respectively. The C atoms of *Hpy* GS and P3P are presented in light blue and yellow.

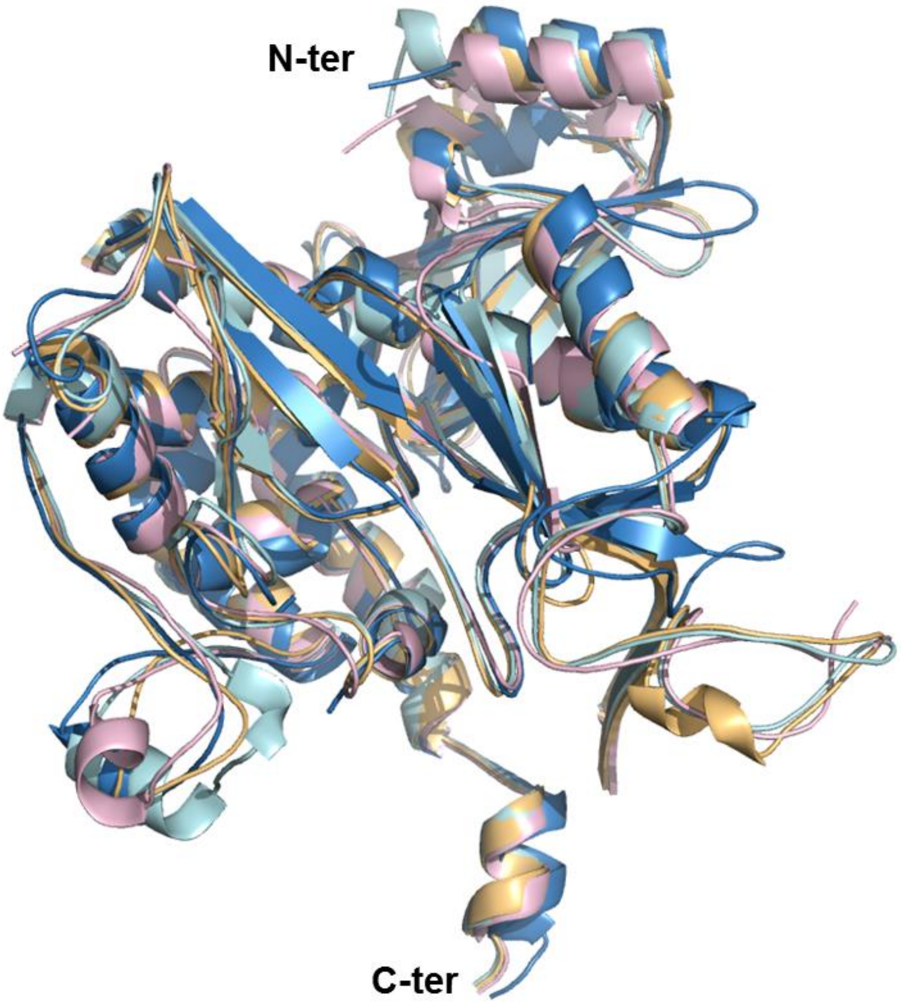

Supplementary Figure 3. Superimpositions of *Hpy* GS with other GS. The *Hpy* GS was superimposed with GS from other organisms with high similarity. GS from *H. pylori*, *S. typhimurium*, *M. tuberculosis*, and *B. hallodurans* are coloured in light blue, light orange, pink, and dark blue, respectively.
